# Limited value of pulse wave analysis in assessing arterial wave reflection and stiffness in the pulmonary artery

**DOI:** 10.14814/phy2.15024

**Published:** 2021-09-23

**Authors:** Junjing Su, Ulf Simonsen, Soren Mellemkjaer, Luke S. Howard, Charlotte Manisty, Alun D. Hughes

**Affiliations:** ^1^ Department of Biomedicine Aarhus University Aarhus Denmark; ^2^ National Heart and Lung Institute Imperial College London London UK; ^3^ Department of Cardiology Aarhus University Hospital Aarhus Denmark; ^4^ MRC Unit for Lifelong Health and Aging Institute of Cardiovascular Science University College London London UK

**Keywords:** arterial stiffness, arterial wave reflection, augmentation index, pulse wave analysis, wave intensity analysis

## Abstract

We explored the use of the augmentation index (AI) based on pulse wave analysis (PWA) in the pulmonary circulation as a measure of wave reflection and arterial stiffness in individuals with and without pulmonary arterial hypertension (PAH) and chronic thromboembolic pulmonary hypertension (CTEPH). Right heart catheterization was performed using a pressure and Doppler flow sensor–tipped catheter to obtain simultaneous pressure and flow velocity measurements in the pulmonary artery in 10 controls, 11 PAH patients, and 11 CTEPH patients. PWA was applied to the measured pressure, while wave intensity analysis (WIA) and wave separation analysis (WSA) were performed using both the pressure and velocity to determine the magnitudes and timings of reflected waves. Type C (AI < 0) pressure waveform dominated in controls and type A (AI > 12%) waveform dominated in PAH patients, while there was a mixture of types A, B, and C among CTEPH patients. AI was greater and the inflection time shorter in CTEPH compared to PAH patients. There was a poor correlation between AI and arterial wave speed as well as measures of wave reflection derived from WIA and WSA. The infection point did not match the timing of the backward compression wave in ~50% of the cases. In patients with type C waveforms, the inflection time correlated well to the timing of the late systolic forward decompression wave caused by ventricular relaxation. In conclusion quantifying pulmonary arterial wave reflection and stiffness using AI based on PWA may be inaccurate and should therefore be discouraged.

## Graphical abstract

We characterized the pressure waveforms in the pulmonary arteries in individuals with and without pulmonary hypertension. Pulse wave analysis (PWA) is unreliable in the pulmonary circulation due to the challenges related to reliable identification of an inflection point and indices derived from PWA may not be indicative of wave reflection or arterial stiffness, particularly when type C pressure waveforms are common. Assessing wave reflection in the pulmonary artery based on PWA should therefore be discouraged.

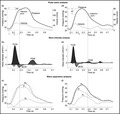

## 1 INTRODUCTION

The main cause of death in patients with pulmonary hypertension (PH) is right heart failure due to increased afterload. In addition to the steady flow resistance, the pulsatile component of the right ventricular load is also important. Arterial pulse pressure is determined by the force of ventricular ejection, arterial compliance and wave reflection; the latter occurs as a consequence of changes in the energy transmission properties of the vessels, e.g., branching or arterial stiffening, causing an admittance mismatch. Reflected waves arriving in the pulmonary artery during systole impose an additional load on the contracting ventricle (Lammers et al., 2012).

**FIGURE 1:**
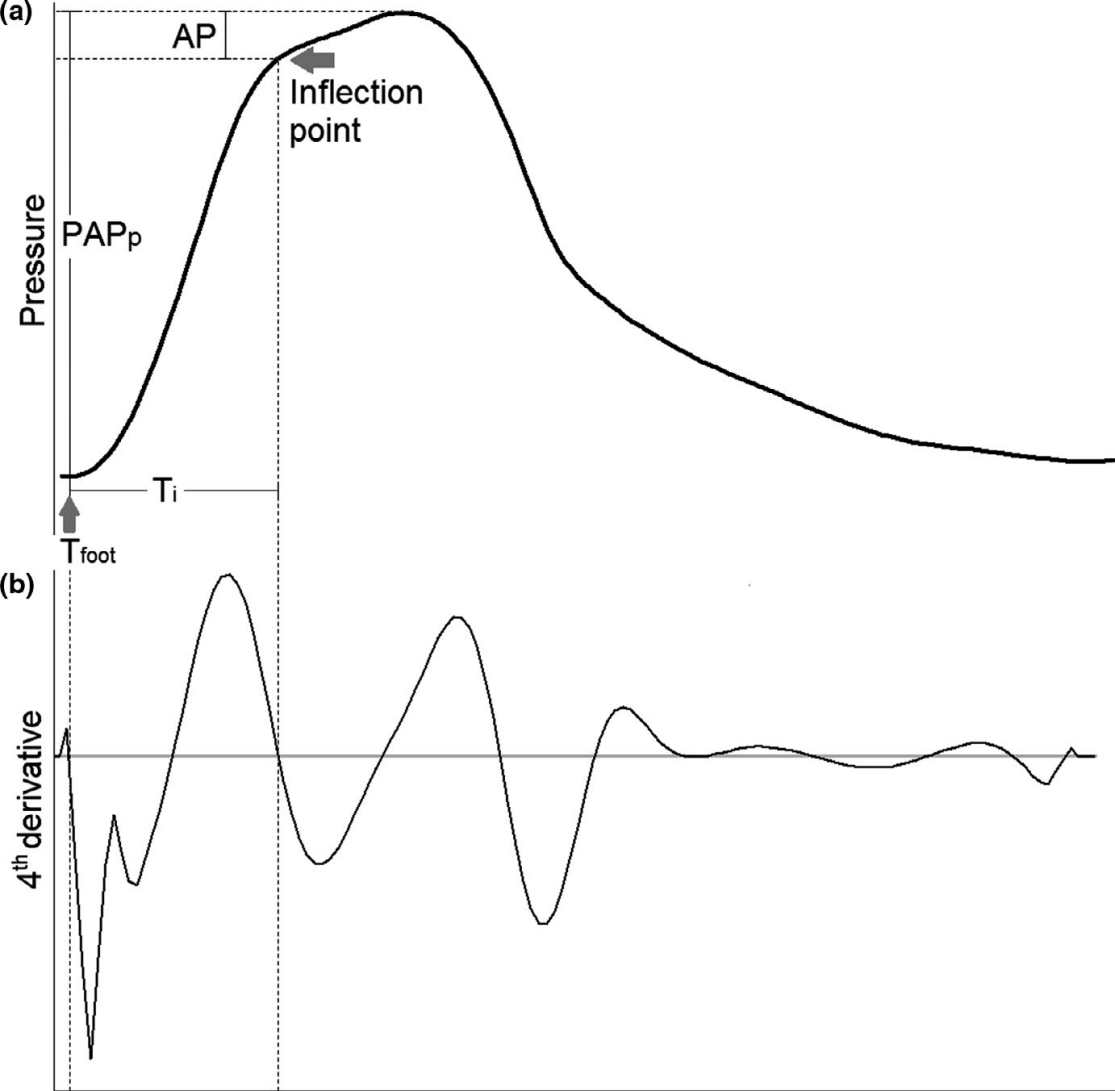
Schematic of pulse wave analysis. In pulse wave analysis (a), the inflection point is identified as the first shoulder of the pressure waveform. The augmentation pressure (AP) is defined as the pressure difference between the inflection on the pressure waveform and the peak systolic pressure and augmentation index (AI) is the ratio of the augmentation pressure to pulmonary arterial pulse pressure (PAP_p_). The inflection time (*T*_i_) was determined as the time difference between the onset of the pressure wave upstroke (*T*_foot_) and the inflection point. The inflection point was determined using the fourth derivative (b) of the ensemble average pressure waveform (Takazawa et al., 1995). The first zero‐crossing from above to below of the fourth derivative was assigned as the onset of the pressure wave upstroke and the inflection point was taken to correspond to the second zero crossing from above to below

Several methods exist to assess the magnitude and timing of arterial wave reflection. One such method is the pulse wave analysis (PWA), which is applied to the measured pressure waveform (Avolio et al., 2009). The augmentation pressure, defined as the pressure difference between the inflection on the pressure waveform in systole and the peak systolic pressure (Figure 1a), corresponds to the part of the pulse pressure that is assumed to be caused by reflected waves and the time of the inflection is interpreted as wave reflection time. As arteries stiffen, there is a faster propagation of the forward pulse wave (increased pulse wave velocity/wave speed) and a more rapid return of reflected waves. Therefore, pressure augmentation and more especially its timing are often considered indirect measures of arterial stiffness. Other time‐domain approaches to assess arterial wave reflection include wave intensity analysis (WIA) and wave separation analysis (WSA), which usually require measurement of pressure as well as flow (velocity). An overview of PWA, WIA, and WSA are outlined in Table 1.

**TABLE 1:** Simple overview of wave reflection as assessed by pulse wave analysis (PWA), wave intensity analysis (WIA) and wave separation analysis (WSA)

	PWA	WIA	WSA
Flow (velocity) data necessary	No	Yes	Yes
Wave speed/impedance calculation necessary	No	Yes/No^a^	Yes
Mathematical approach	Identifies the second zero‐crossing of the pressure derivative	Calculates the product of the temporal changes in pressure and velocity	Decomposes measured pressure and flow into forward and backward components
Reflected waves appear as	Augmentation pressure (AP, unit: mmHg)	Backward compression (decompression) wave (BCW/BDW, unit: W/m^2^ & J/m^2^)	Backward pressure or flow (*P*_b_ & *Q*_b_, unit: mmHg & m^3^/s)
Quantification of wave reflection	Augmentation index (AI): ratio of augmentation to pulse pressure	Wave reflection index (WRI): Ratio of BCW (BDW) to forward compression wave energy	*P*_b_/*P*_f_ or *Q*_b_/*Q*_f_: ratio of backward to forward pressure or flow

^a^ Wave speed calculation is necessary for wave intensity separation, but not for the calculation of net wave intensity.

The usefulness of PWA as a complementary investigation to conventional peripheral blood pressure measurement in managing systemic arterial hypertension and predicting cardiovascular events has been demonstrated in several large studies (Agabiti‐Rosei et al., 2007; Chirinos et al., 2012; Vlachopoulos et al., 2010; Weber et al., 2005). In contrast, application of PWA in the pulmonary circulation is limited, partly due to the lack of non‐invasive approaches; therefore, the clinical relevance of PWA in pulmonary hypertension is unclear and warrants investigation. Two well‐referenced studies (Castelain et al., 2001; Nakayama et al., 2001) published in 2001 have documented that earlier inflection time and greater pressure augmentation are present in patients with chronic thromboembolic pulmonary hypertension (CTEPH) compared to patients with pulmonary arterial hypertension (PAH). This was interpreted as earlier and larger wave reflection in patients with CTEPH and it has been suggested that PWA may be useful in the differential diagnosis of CTEPH and PAH. However, wave reflection affects both the arterial pressure and flow and the validity of pressure augmentation as a measure of wave reflection has been questioned in the systemic circulation (Hughes, Park, et al., 2013). Furthermore, a previous study by our group comparing wave reflection using wave analyses that are based on both arterial pressure and flow measurement found similar degree of wave reflection between CTEPH and PAH (Su et al., 2017). We therefore hypothesized that quantifying pulmonary arterial wave reflection and stiffness using AI based on PWA may be inaccurate.

Here, we aimed to characterize the pressure waveforms in subjects without pulmonary vascular disease and patients with PAH and CTEPH and assess the limitations of PWA in the pulmonary circulation. Furthermore, we compared the reflection parameters derived from PWA to parameters derived from WIA and WSA.

## 2 METHODS

### 2.1 Data acquisition

Control subjects without known pulmonary vascular disease, PAH patients and CTEPH patients were selected among patients undergoing cardiac catheterization for clinical reasons at Hammersmith Hospital, Imperial College Healthcare and Aarhus University Hospital. Only CTEPH patients that were suitable for surgery were included as these patients were considered to have more proximal vascular lesions and provided a better contrast with PAH patients. Details on patient recruitment have been published previously (Su et al., 2017, 2019). The study conformed to the Declaration of Helsinki and was approved by the local Ethics Committees (references13/LO/1305 and M‐2013‐278‐13, respectively) and all participants gave written informed consent.

As described previously (Su et al., 2017), right heart catheterization was performed using a 6 Fr multipurpose catheter or a 6 Fr balloon flotation catheter that was advanced into the pulmonary artery (PA) via the right femoral, brachial or internal jugular vein. A combined dual‐tipped pressure and Doppler flow sensor wire (Combowire, Philips Volcano) was then advanced approximately 1 cm beyond the end of the catheter. Careful manipulation of the catheter and wire ensured that the pressure and Doppler flow velocity signals were optimized in situ. Once stable signals were observed, pressure and velocity data were acquired simultaneously (Combomap, Philips Volcano) at a sampling rate of 200 Hz for 30–60 s together with ECG monitoring. Data were collected from the main PA and either the left or right PA (here after referred to as branch PA) in controls and PAH patients. In CTEPH patients, data were collected from the main and right PAs and the measurements were repeated 3 months after pulmonary endarterectomy (PEA; Su et al., 2019).

### 2.2 Pulse wave analysis

Pulse wave analysis, WIA, and WSA were performed using custom‐written Matlab software (MathWorks). Pressure and velocity signals were ensemble‐averaged using the *R*‐wave on the ECG as a fiducial marker. In PWA, the inflection point, defined as the first shoulder of the pressure waveform, was determined using the fourth derivative of the ensemble average pressure waveform (Figure 1b) as previously described (Takazawa et al., 1995). The first zero‐crossing from above to below of the fourth derivative was assigned as the onset of the pressure wave upstroke and the shoulder was taken to correspond to the second zero crossing. The magnitude of the secondary rise in pressure (from the shoulder to the peak) is the augmentation pressure (Figure 1a). Augmentation index (AI) was calculated as the ratio of the augmentation pressure to arterial pulse pressure. AI > 50% and negative AI were considered implausible as a measure of wave reflection so additional analyses excluding 0 ≤ AI ≤ 50% were also pre‐specified (Baksi et al., 2016; Hughes, Park, et al., 2013). In accordance to Murgo et al. (Murgo et al., 1980), pressure waveforms were classified into types A (AI > 12%), B (0 ≤ AI ≤ 12%) and C (AI < 0, i.e., where the peak systolic pressure preceded a well‐defined inflection point). The inflection time (*T*_i_) was determined as the time difference between the foot of the upslope of the pressure waveform and the inflection point.

### 2.3 Wave intensity and wave separation analyses

Wave intensity analysis was performed as described previously with the values normalized to cardiac cycle length to make it independent of sampling rate (Su et al., 2017). The net wave intensity is the product of the change in pressure (*dP*) and velocity (*dU*). The local wave speed (*c*), a measure of pulmonary arterial stiffness was calculated using the sum of squares method (Equation 1; Davies et al., 2006). With the knowledge of local wave speed, wave intensity was separated into its forward (WI_+_) and backward (WI_–_) components (Equation 2; Su et al., 2017); and the measured pressure was separated into forward (*P*_f_) and backward (*P*_b_) pressures in accordance with WSA (Equation 3; Hughes et al., 2013).

$$c=\frac{1}{\rho }\cdot \sqrt{\frac{\sum dP^{2}}{\sum dU^{2}}},\left(1\right)$$

$${\text{WI}}_{\pm }=\pm {\left(\frac{\mathrm{dP}}{\mathrm{dt}}\pm \rho c\cdot \frac{\mathrm{dU}}{\mathrm{dt}}\right)}^{2}\cdot {\text{CCD}}^{2}/\left(4\rho c\right),\left(2\right)$$

$$P_{f/b}=\frac{1}{2}\cdot \left(P\pm \rho cU\right),\left(3\right)$$

*ρ* is the blood density, assumed to be 1040 kg/m^3^ and the sum is taken over one cardiac period and CCD is the duration of the cardiac cycle.

Separated waves derived from WIA were quantified by the cumulative area under each wave (J/m^2^) corresponding to the energy density over a cardiac cycle squared. Peak *P*_f_ and *P*_b_ were quantified after subtracting the diastolic pressure. Wave reflection was quantified by the wave reflection index (WRI), defined as the ratio of the backward compression wave (BCW) to forward compression wave (FCW) energy, and by the ratio of peak *P*_b_ to *P*_f_. The timing of peak BCW (*T*_BCW_), peak forward decompression wave (*T*_FDW_) and peak *P*_b_ (*T*_Pb_) were determined with respect to the foot of the measured pressure. The travel time of the reflected wave (Δ*T*) was defined as the time difference between the peak of FCW to BCW.

### 2.4 Statistical analysis

Results are expressed as mean ± *SD* or median (25%–75% quartile) as appropriate. Differences between the controls, PAH patients and pre‐PEA CTEPH patients were compared using one‐way ANOVA (or the Kruskal Wallis test for non‐parametric data and groups with *n* < 5) followed by a Bonferroni test (or a Dunn's test) to control the familywise error rate. Chi‐square tests and Fisher's exact tests were used for categorical data. Student's *t* tests or Wilcoxon's rank‐sum test (non‐parametric data and groups with *n* < 5) were used to assess differences between post‐PEA CTEPH patients and the control group. Paired Student's *t* test or the Wilcoxon signed‐rank test were used to compare differences between the data from the main and branch PAs and between pre‐ and post‐PEA data in CTEPH patients. The relationship between variables were examined by Pearson's correlation or, where appropriate, Spearman's correlation analysis. Strength of association was estimated by calculating the standardized beta‐coefficient derived from a multilevel regression analysis with disease category and individuals as random effects. Bland‐Altman plots were used to assess the agreement between the inflection time and *T*_BCW_ and *T*_FDW_. The level of significance was set at *p* < 0.05. All statistical analyses were performed using Stata 13 (StataCorp).

## 3 RESULTS

### 3.1 Pressure waveforms

Participant characteristics are shown in Table 2. Analyses were carried out in 10 control subjects (59 ± 14 years, 8 male), 11 PAH patients (56 ± 21 years, 2 male) and 11 operable CTEPH patients (64 ± 10 years, 3 male). There were no marked differences in age, height, and body surface area between the three groups. Eight of the CTEPH patients completed the 3‐month follow‐up investigations post‐PEA.

**TABLE 2:** Participant characteristics

	Control (*N* = 10)	PAH (*N* = 11)	Pre‐PEA (*N* = 11)	Post‐PEA (*N* = 8)
Male, *n* (%)	8 (80%)	2 (18%)	3 (27%)	3 (38%)
Age, years	59 ± 14	56 ± 21	64 ± 10	67 ± 9
Height, m	1.7 ± 0.1	1.6 ± 0.1	1.7 ± 0.1	1.7 ± 0.1
Weight, kg	81 ± 16	73 ± 12	82 ± 21	76 ± 19
Heart rate, min^−1^	73 ± 8	81 ± 8	80 ± 14	80 ± 11
Cardiac index, L min^−1^ m^−2^	2.6 ± 0.5	2.3 ± 1.1	2.2 ± 0.7	2.8 ± 0.9^†^
PAPm, mmHg	17 ± 3	47 ± 11*	48 ± 10*	32 ± 13*
Systolic PAP, mmHg	26 ± 3	76 ± 16*	82 ± 14*	55 ± 22*, ^,†^
PAPp, mmHg	13 ± 2	43 ± 10*	51 ± 10*	33.8 ± 15.1*, ^,†^
PAPp/PAPm ratio	0.8 ± 0.2	0.9 ± 0.2	1.1 ± 0.2*	1.1 ± 0.3*
PAWP, mmHg	—	9 ± 2	10 ± 3	10 ± 3
Indexed TPR, Wood units/m^2^	7.0 ± 2.0	24.6 ± 12.8*	24.5 ± 9.1*	13.5 ± 9.0*, ^,†^
Compliance, ml/mmHg	5.3 ± 1.6	1.3 ± 0.8*	1.1 ± 0.3*	2.2 ± 0.9*, ^,†^

Note

Detailed participant characteristics have been reported previously (Su et al., 2017, 2019).

Abbreviations: PAH, pulmonary arterial hypertension; PAPm, mean pulmonary arterial pressure; PAPp, pulmonary arterial pulse pressure; PAWP, pulmonary arterial wedge pressure; PEA, pulmonary endarterectomy; TPR, total pulmonary resistance.

* *p* < 0.5 vs control.

† *p* < 0.5 vs pre‐PEA.

**FIGURE 2:**
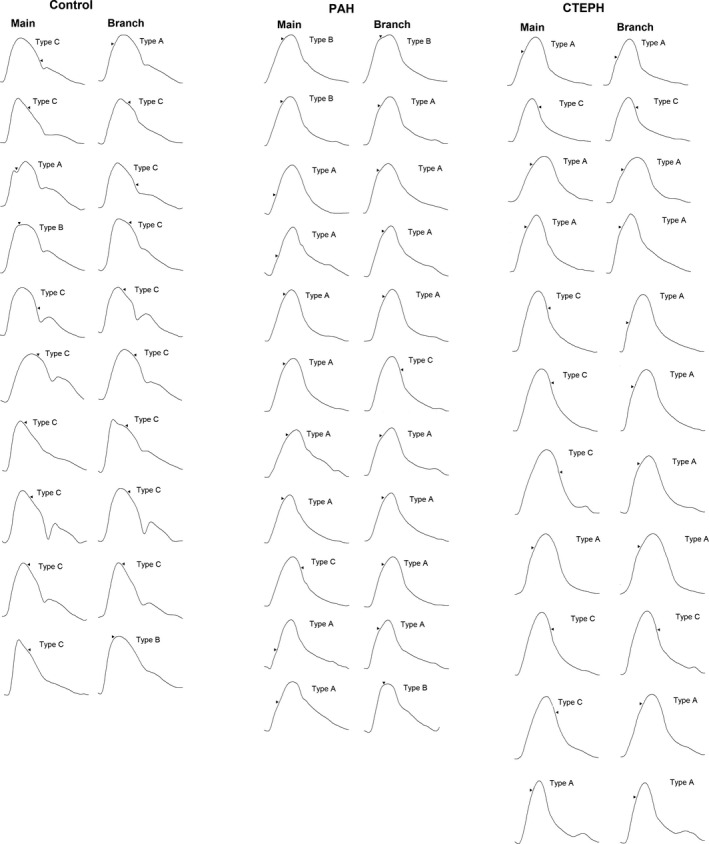
Pressure waveforms of all individuals. The inflection point (hollow triangle) was determined as the second zero crossing of the fourth derivative of the ensemble average pressure waveform. Pressure waveforms were classified into types (a) (augmentation index [AI] >12%), (b) (0 ≤ AI ≤ 12%) and (c) (AI < 0). CTEPH, chronic thromboembolic pulmonary hypertension; PAH, pulmonary arterial hypertension

The morphology of the pressure waveforms of the controls differed from the PH patients as shown by the more obvious dicrotic notch and the more convex downslope, contrasting with the rapid downstroke of the PH patients. In approximately 50% of the participants, a discernible inflection point could be identified (pressure waveforms of all participants are shown in Figure 2). In other cases, the upslopes and downslopes of the pressure traces were smooth and monotonic; therefore, it was difficult to determine a convincing inflection point; this was especially common in controls and the main PA of the PH patients. We did not observe any difference in the pressure waveforms between the left and right PAs

**FIGURE 3:**
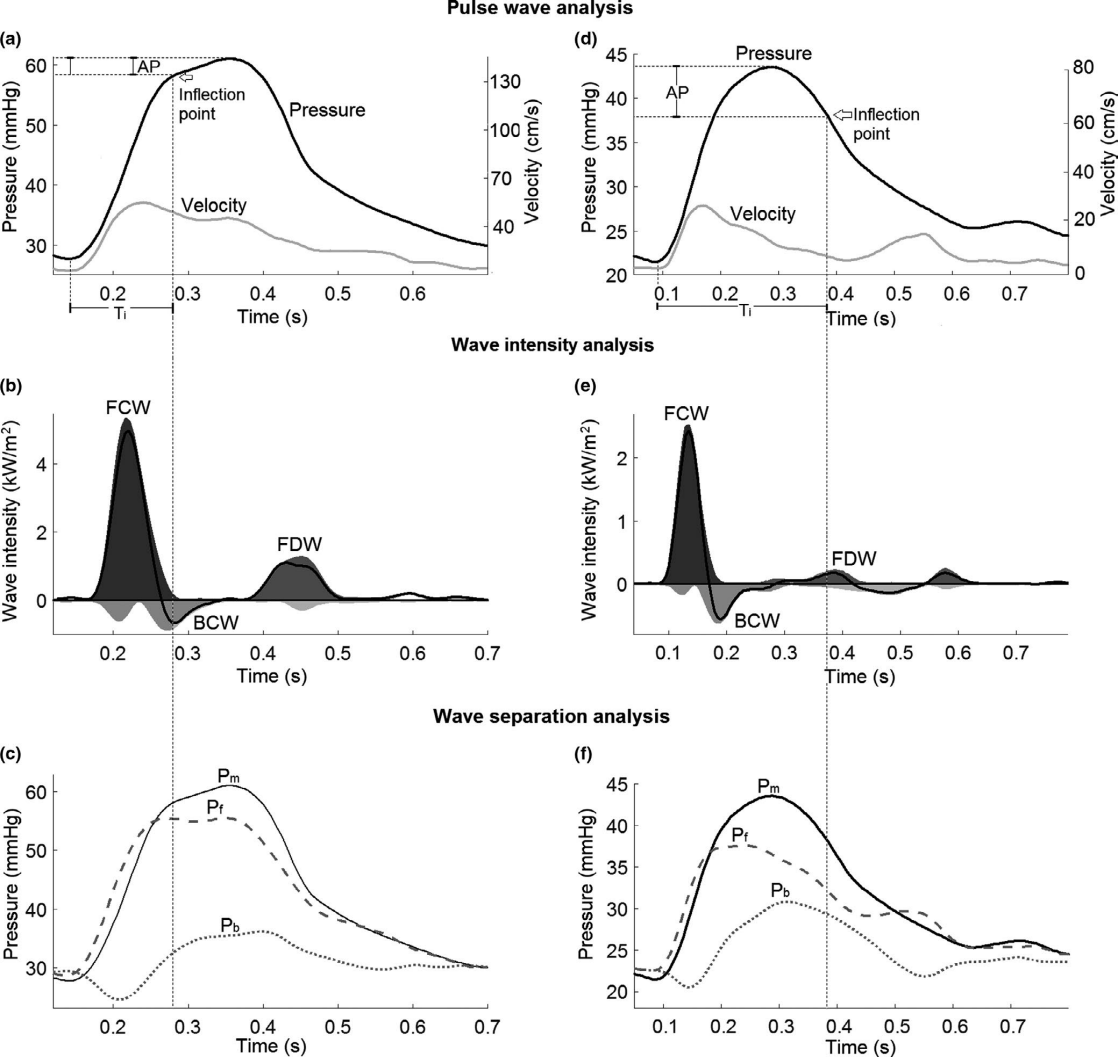
Examples of arterial wave analyses. Arterial wave analyses of two pulmonary hypertension patients (a–f, respectively) are shown. In pulse wave analysis (a and d), the effect of the reflected waves is conventionally provided by the augmentation index (AI), i.e., the ratio of the augmentation pressure (AP) to pulmonary arterial pulse pressure. Wave intensity analysis (b and e) of the same patients revealed an initial forward compression wave (FCW) related to right ventricular contraction, a late systolic forward decompression wave (FDW) related to ventricular relaxation, and a mid‐systolic backward compression wave (BCW) due to wave reflection. The contour of the net wave intensity is highlighted in black. Finally, wave separation analysis (c and f) separates the measured pressure (*P*_m_) into a forward (*P*_f_) and backward (reflected) pressure (*P*_b_). Here, we show an example of an easily identifiable inflection point (a), where the inflection time (*T*_i_) corresponds to a decrease in velocity and coincides with the timing of BCW (b) and the increasing *P*_b_ (c). In another case, a convincing inflection point was difficult to identify (d) due to the smooth contour of the pressure waveform. The late inflection point detected after the peak systolic pressure did not correspond to the sudden decrease in the velocity in early systole or the timings of BCW (e) and *P*_b_ (*F*), although it coincided with the timing of FDW

Illustrations of PWA, WIA, and WSA are shown in Figure 3. We show an example of an easily identifiable shoulder on the pressure waveform (Figure 3a), where the inflection point corresponded to a decrease in velocity and coincided with the timings of BCW and the increasing *P*_b_ (Figure ). In another case, a convincing inflection point was not evident (Figure 3d) due to the smooth contour of the pressure waveform. The late inflection point detected on the pressure waveform did not correspond to the sudden decrease in velocity in early systole or the timings of BCW and *P*_b_ (Figure 3e,f).

The majority of the controls displayed type C pressure waveforms both in the main and branch PAs, while type A waveforms dominated in PAH patients (Table 3; Figure 2). Among pre‐PEA CTEPH patients, approximately half displayed type C and the other half type A pressure waveforms in the main PA (Table 3; Figure 2), while type A waveforms dominated in the branch PA. Following PEA, type B pressure waveform dominated in the main and type C waveform dominated in the branch PA.

**TABLE 3:** Pressure waveform types

	Control	PAH	Pre‐PEA	Post‐PEA
Main pulmonary artery				
Type A (AI > 12%)^a^	1 (10%)	8 (73%)	5 (45%)	2 (25%)
Type B (0 ≤ AI ≤ 12%)	1 (10%)	2 (18%)	0	4 (50%)
Type C (AI < 0%)	8 (80%)	1 (9%)	6 (55%)	2 (25%)
	*χ*^2^ = 12.5, *p* = 0.02; Fisher's *p* = 0.004			
Branch pulmonary artery				
Type A^a^	1 (10%)	8 (73%)	9 (82%)	2 (25%)
Type B	1 (10%)	2 (18%)	0	1 (12.5%)
Type C	8 (80%)	1 (9%)	2 (18%)	5 (62.5%)
	*χ*^2^ = 16.5, *p* = 0.002; Fisher's *p* = 0.001			

Note

Data are presented as *n* (%). Pressure waveforms were classified into types A, B and C as described by Murgo et al., (1980).

Abbreviations: AI, augmentation pressure; PAH, pulmonary arterial hypertension; PEA, pulmonary endarterectomy.

^a^ Among patients with type A pressure waveform, three PAH and two post‐PEA CTEPH patients had an AI > 50% in the main pulmonary artery and one pre‐PEA CTEPH patient had an AI > 50% in the branch pulmonary artery.

### 3.2 Augmentation index and inflection time

**FIGURE 4:**
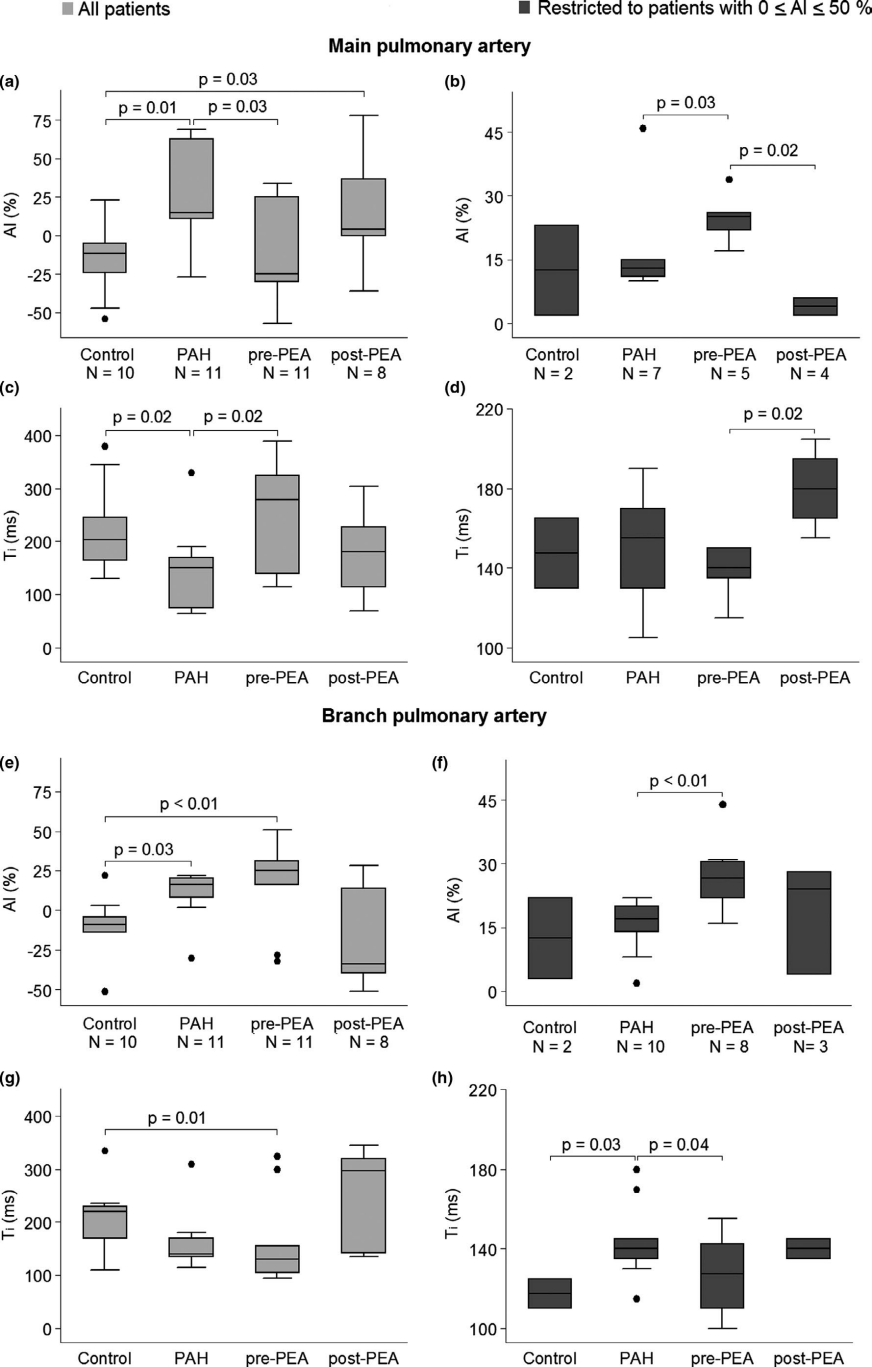
Group comparison of augmentation index and inflection time. Comparison of the augmentation index (AI) and inflection time (*T*_i_) in the main (a–d) and branch (e–h) pulmonary arteries between controls, patients with pulmonary arterial hypertension (PAH) and patients with chronic thromboembolic pulmonary hypertension (CTEPH) before and after pulmonary endarterectomy (PEA). In the left panel, all participants are included and in the right panel, only participants with 0 ≤ AI ≤ 50% are included. Differences between the groups were compared using the Kruskal Wallis test or the Wilcoxon rank‐sum test. Paired data were analyzed using the Wilcoxon signed‐rank test

Inter‐group comparison (Figure 4a) showed that in the main PA, AI was greater in the PAH group compared to the controls and the CTEPH (pre‐PEA) group, consistent with the dominance of type A pressure waveform in the PAH group. *T*_i_ was also shorter (Figure 4c) in the PAH group. In the branch PA (Figure 4e,g), AI was greatest in the CTEPH (pre‐PEA) group and lowest in the control group. Correspondingly, *T*_i_ was shortest in the CTEPH group. After excluding subjects with AI > 50% and subjects with type C pressure waveforms (i.e., nearly all control subjects) in both the main and branch PAs, AI was significantly greater (Figure 4b,f) in CTEPH (pre‐PEA) patients compared to PAH patients. *T*_i_ was significantly shorter (Figure 4d,h) in CTEPH patients compared to PAH patients in the branch PA, but not in the main PA.

Following PEA (Figure 4), there was no evidence of change in AI and *T*_i_ in the main PA, while AI was lower and *T*_i_ longer in the branch PA compared to pre‐PEA values, but the differences did not reach statistical significance. After restricting to patients with 0 ≤ AI ≤ 50%, AI was significantly lower and *T*_i_ significantly longer in the main PA post‐PEA, while the differences in the branch PA remained statistically insignificant. AI of post‐PEA individuals was greater compared to controls (Figure 4a).

### 3.3 Wave intensity and wave separation analyses

Wave intensity analysis revealed three distinctive systolic waves in PH patients (Figure 3b,e): an initial FCW related to ventricular contraction followed by a mid‐systolic BCW attributed to reflection of the FCW and a forward decompression wave (FDW) in late systole caused by ventricular relaxation. In controls, the intensity of the backward travelling wave was minimal. As has been reported in detail previously (Su et al., 2017), BCW and WRI were significantly greater in PAH and CTEPH (pre‐PEA) patients compared to controls (Table 4). Similarly, WSA revealed a significantly higher peak *P*_b_ and greater *P*_b_/*P*_f_ in PAH and CTEPH patients compared to controls (Table 4). The differences in WRI and *P*_b_/*P*_f_ between PAH and CTEPH patients were not statistically significant. While BCW energy and WRI were similar in the main and branch PAs, *P*_b_ and *P*_b_/*P*_f_ were greater in the branch PA compared to the main PA in PAH and CTEPH patients. Δ*T*, i.e., the time interval between peak FCW and BCW was longer, and the timing of the peak *P*_b_ (*T*_Pb_) was delayed in the main PA in CTEPH (pre‐PEA) patients compared to controls and PAH patients.

**TABLE 4:** Wave reflection as assessed by wave intensity and wave separation analyses

		Control (*n* = 7)	PAH (*n* = 11)	Pre‐PEA (*n* = 11)	Post‐PEA (*n* = 8)
**Main pulmonary artery**					
WIA	Wave speed	3.12 (2.69–3.84)	11.9 (8.4–13.3)*	15.1 (11.5–16.8)*	5.76 (5.37–9.19)*^,^, ^
	BCW energy (kJ/m^2^)	0.17 (0.14–0.21)	1.51 (0.82–1.80)*	2.36 (0.73–3.37)*	1.00 (0.70–2.00)*
	WRI (%)	3.82 (3.33–5.83)	25.1 (19.3–29.6)*	30.2 (11.8–38.8)*	21.2 (16.2–25.9)*
	ΔT (ms)	55 (45–100)	63 (55–85)	100 (65–125)*, ^,†^	68 (50–80)
WSA	*P*_b_ (mmHg)	2 (2–4)	13 (10–17)*	14 (10–19)*	7 (5–10)*
	*P*_b_/*P*_f_ (%)	23.0 (13.8–29.3)	43.0 (28.4–50.0)*	38.7 (23.0–54.6)	35.7 (19.3–42.5)
	*T*_Pb_ (ms)	200 (115–280)	223 (205–235)	255 (230–275)*, ^,†^	258 (215–280)
**Branch pulmonary artery**					
WIA	Wave speed	2.29 (2.06–3.00)	13.3 (8.2–14.9)*	15.7 (11.5–20.9)*	8.11 (7.75–10.91)*^,^, ^
	BCW energy (kJ/m^2^)	0.20 (0.16–0.45)	1.70 (1.06–2.02)*	1.64 (1.22–1.89)*	1.23 (0.82–1.46)*
	WRI (%)	3.92 (2.65–8.86)	24.7 (18.9–32.6)*	29.1 (25.2–36.6)*	20.8 (13.4–29.3)*
	Δ*T* (ms)	65 (45–95)	60 (55–65)	83 (65–90)^†^	75 (58–95)
WSA	*P*_b_ (mmHg)	3 (2–4)	18 (15–21)*, ^,‡^	24 (16–25)*, ^,‡^	12 (8–18)*^,^, ^
	*P*_b_/*P*_f_ (%)	25.5 (21.0–37.1)	53.0 (46.1–66.0)*, ^,‡^	57.2 (48.6–63.8)*, ^,‡^	44.9 (34.2–63.6)*
	*T*_Pb_ (ms)	233 (148–295)	220 (210–250)	238 (220–250)	243 (223–253)

Note

Data are presented as median (25%–75% quartile). Control subjects with mid‐systolic backward decompression waves or negative *P*_b_ were excluded.

BCW and WRI have been published previously (Su et al., 2017, 2019).

Abbreviations: BCW, backward compression wave; PAH, pulmonary arterial hypertension; *P*_b_, backward pressure; PEA, pulmonary endarterectomy; *P*_f_, forward pressure; *T*_Pb_, arrival time of peak *P*_b_; WRI, wave reflection index; Δ*T*, time interval from the peak of forward compression wave to peak BCW.

* *p* < 0.05 compared to control.

† *p* < 0.05 compared to PAH.

‡ *p* < 0.05 compared to main PA.

^ *p* < 0.05 compared to pre‐PEA.

Following PEA, *P*_b_ decreased, consistent with an overall decrease in pulmonary pressures. The decrease in BCW energy, WRI and *P*_b_/*P*_f_ were small and not statistically significant (Table 4). When compared to controls, BCW energy, WRI, *P*_b_ and *P*_b_/*P*_f_ of post‐PEA CTEPH patients remained greater.

### 3.4 Comparing PWA to WIA and WSA

**FIGURE 5:**
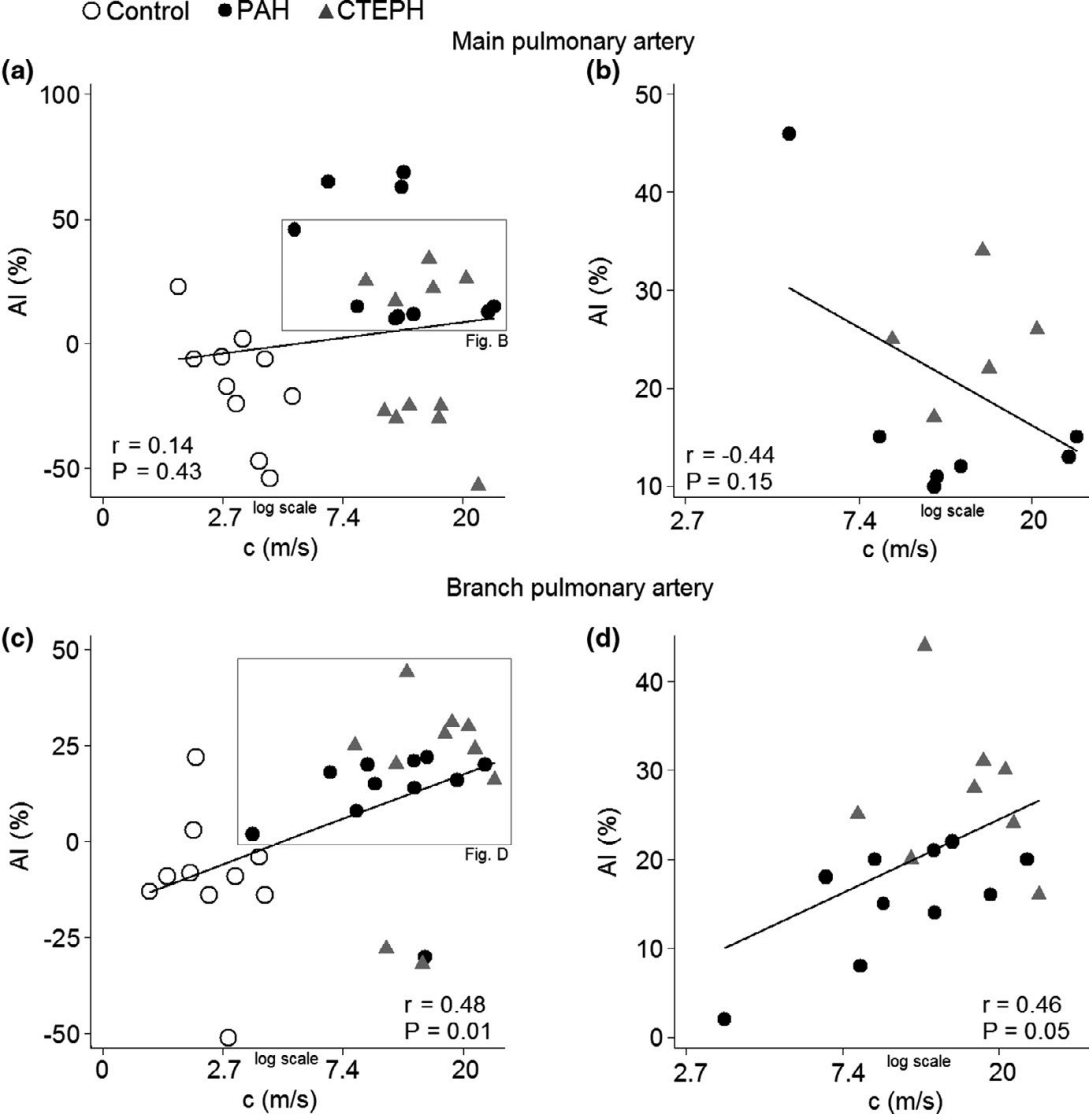
Correlation between augmentation index and wave speed. The augmentation index (AI) was poorly correlated to the wave speed (c) in the main pulmonary artery, while there was a moderate correlation in the branch pulmonary artery. In the left panel (a and c), all participants are included; in the right panel (b and d), only patients with pulmonary arterial hypertension (PAH) and chronic thromboembolic pulmonary hypertension (CTEPH) that have 0 ≤ AI ≤ 50% are included

Comparisons between the parameters derived from PWA, WIA, and WSA were conducted in all participants followed by subgroup analysis restricted to PH patients with 0 ≤ AI ≤ 50% (controls were not included, as they differed substantially from the PH patients). AI correlated poorly with the local arterial wave speed in the main PA (Figure 5a,b), while there was a moderate and significant correlation in the branch PA (Figure 5c,d). *T*_i_ showed a negligible correlation with the local wave speed in the main PA (overall: *r* = −0.07, *p* = 0.71; PH patients with 0 ≤ AI ≤ 50%: *r* = 0.26, *p* = 0.26) and a weak negative correlation in the branch PA (overall: *r* = −0.36, *p* = 0.05; PH patients with 0 ≤ AI ≤ 50%: *r* = −0.21, *p* = 0.35).

**FIGURE 6:**
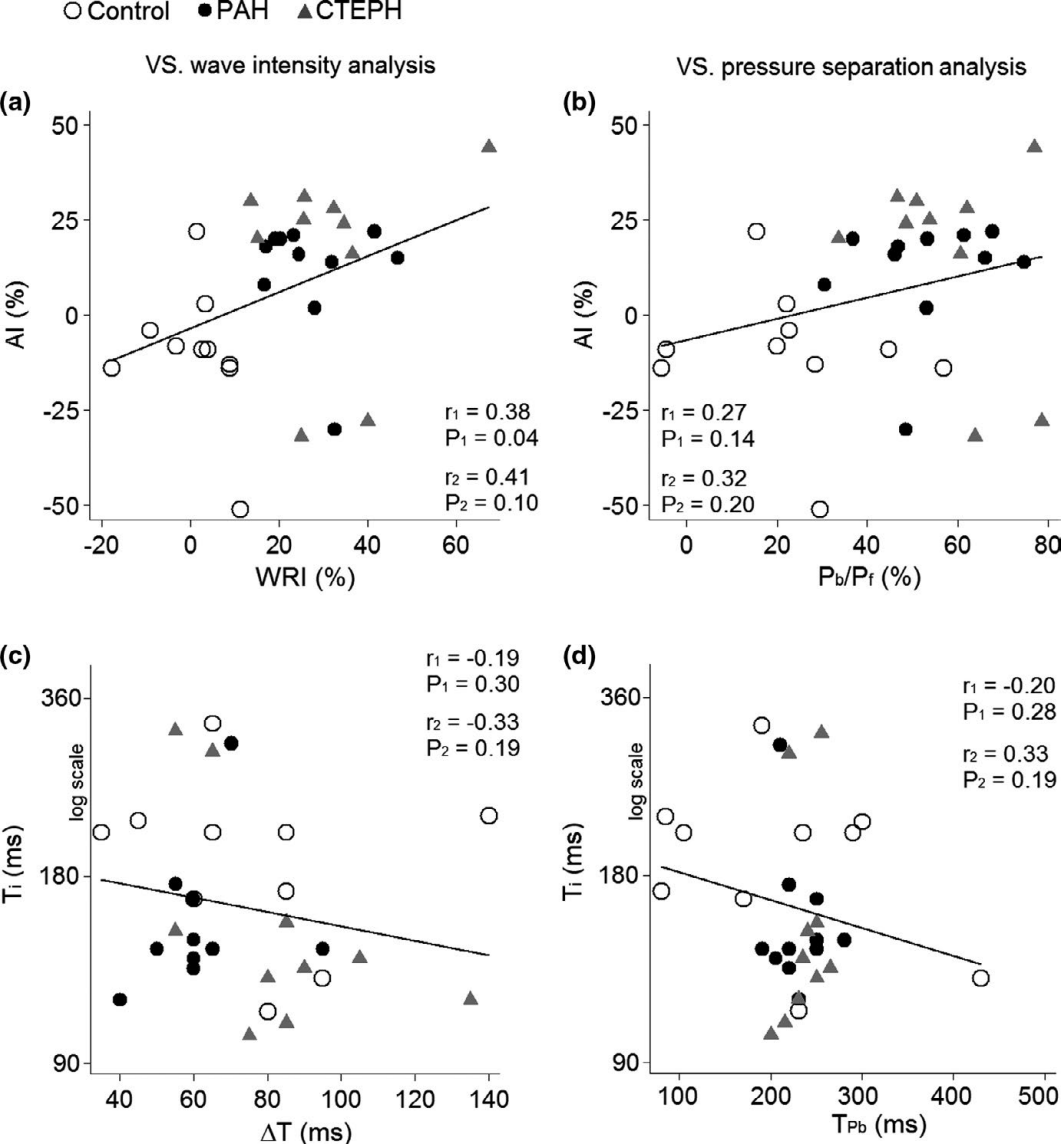
Simple correlation analyses of wave reflection magnitudes and timings. The augmentation index (AI) was poorly correlated to wave reflection index (WRI, a) and the ratio of backward to forward pressure (*P*_b_/*P*_f_, b). The inflection time (*T*_i_) was poorly correlated to the traveling time of the backward compression wave (Δ*T*, c) and the timing of the peak backward pressure (*T*_Pb_, d). Data are from the branch pulmonary artery of all participants. The two Pearson correlation coefficients given are for all participants (*r*_1_) and for patients with pulmonary arterial hypertension (PAH) and chronic thromboembolic pulmonary hypertension (CTEPH) that have 0 ≤ AI ≤ 50% (*r*_2_). Similar findings were observed in the main pulmonary artery

There was a weak, but statistically significant correlation between AI and WRI in the branch PA (Figure 6a), but not the main PA (*r* = 0.21, *p* = 0.25), while AI was poorly correlated to *P*_b_/*P*_f_ in the main (*r* = 0.23, *p* = 0.22 and branch PA, Figure 6b). Also, *T*_i_ was poorly correlated to Δ*T* (main PA: *r* = 0.08, *p* = 0.67), *T*_BCW_ (main PA: *r* = −0.09, *p* = 0.63) and *T*_Pb_ (main PA: *r* = 0.25, *p* = 0.18) in both the main and branch PA (Figure 6c,d). In contrast, *P*_b_/*P*_f_ was strongly and significantly correlated to WRI (main PA: *r* = 0.73, *p* < 0.01, branch PA: *r* = 0.84, *p* < 0.01) and it remained so when restricted to patients with 0 ≤ AI ≤ 50%. *T*_Pb_ was poorly correlated to Δ*T* and (main PA: rho = −0.06, *p* = 0.73; branch PA: rho = −0.29, *p* = 0.11) and T_BCW_ (main PA: rho = −0.05, *p* = 0.81; branch PA: rho = −0.08, *p* = 0.67). Examining the relationship between the parameters derived from PWA, WIA, and WSA using a multilevel regression model yielded similar results.

**FIGURE 7:**
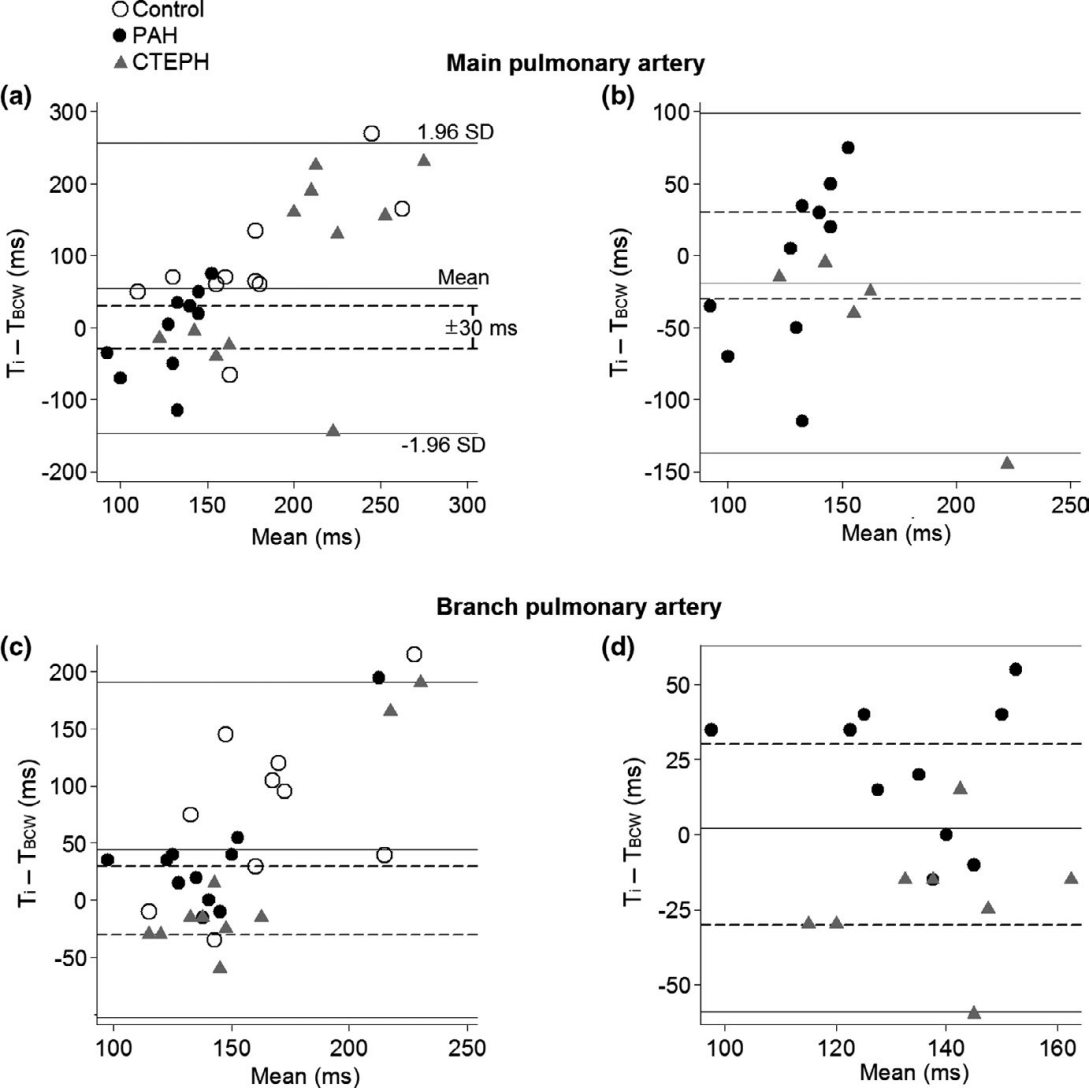
Bland–Altman plots of the inflection time (*T*_i_) and timing of the peak backward compression wave (*T*_BCW_). In the left panel (a, c), all participants are included. In the right panel (b, d), only patients with pulmonary arterial hypertension (PAH) and chronic thromboembolic pulmonary hypertension (CTEPH) that have 0≤ augmentation index ≤50% are included. Note that the errors in the plots are not uniform

Bland Altman plots revealed a wide limit of agreement between the inflection time and the timing of peak BCW (Figure 7). *T*_i_ coincided closely with T_BCW_ (±30 ms) in 19% (main PA) and 45% (branch PA) of the cases (Figure 7a,c). When limited to PH patients with 0 ≤ AI ≤ 50%, *T*_i_ coincided with *T*_BCW_ in 50% (main PA) and 67% (branch PA) of the patients (Figure 7b,d).

**FIGURE 8:**
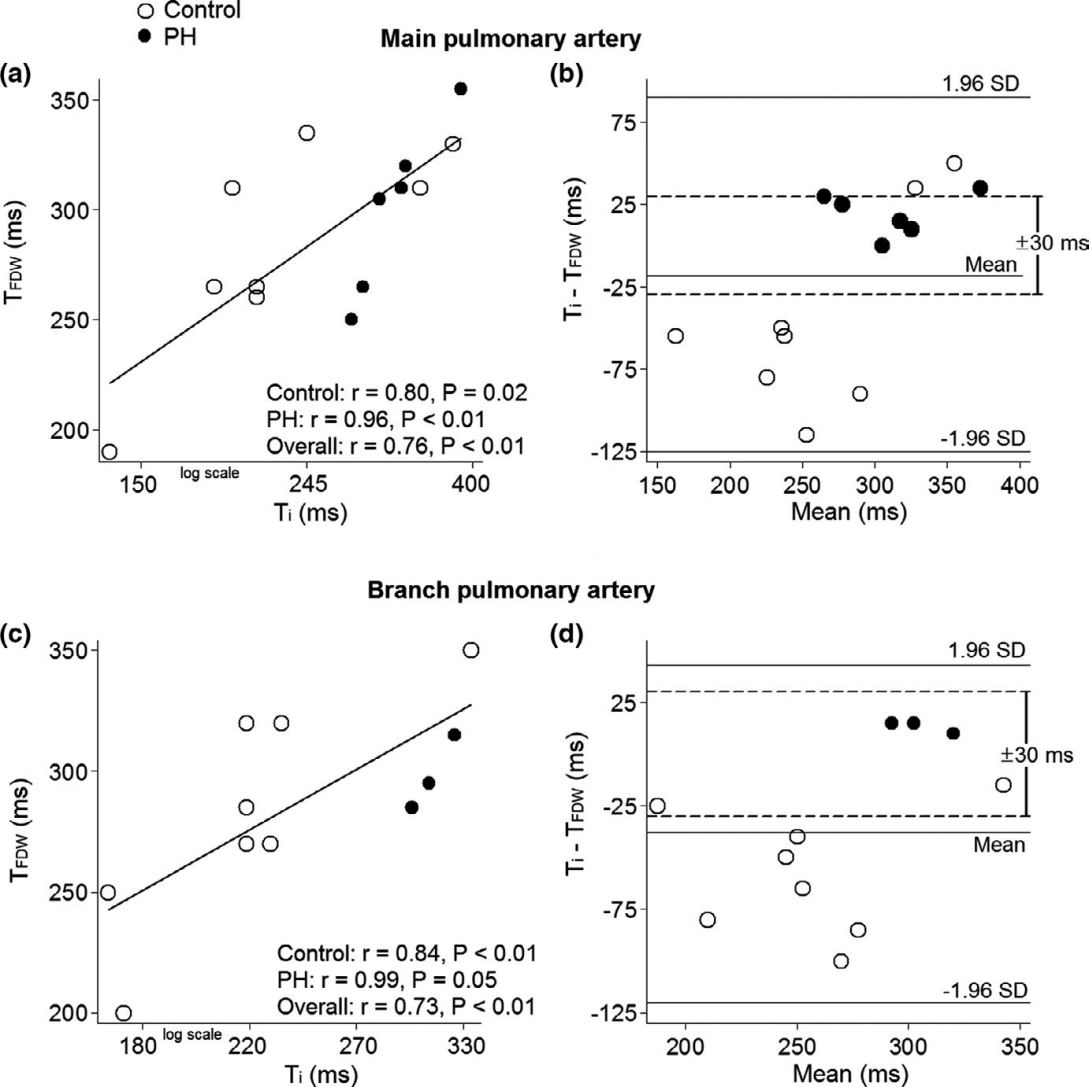
Exploring the inflection point of type C pressure waveforms. In individuals with type C pressure waveforms, the detected inflection time (*T*_i_) was closely correlated to the timing of the peak forward decompression wave (*T*_FDW_, a and c). In pulmonary hypertension (PH) patients, *T*_i_ matched *T*_FDW_ (±30 ms), while *T*_i_ arrived earlier than FDW in controls (b and d)

Among participants (controls and PH patients) with type C pressure waveforms, the inflection time correlated poorly to *T*_BCW_ (main PA: rho = 0.34, *p* = 0.24; branch PA: rho = 0.28, *p* = 0.40) and Δ*T* (main PA: rho = 0.26, *p* = 0.36; branch PA: rho = −0.02, *p* = 0.96). However, it was strongly and significantly correlated to the timing of peak FDW (Figure 8a,c). Although there was a wide limit of agreement, *T*_i_ coincided with T_FDW_ (±30 ms) in the PH patients, while in control subjects, the inflection point generally appeared earlier than *T*_FDW_ (Figure 8b,d).

## 4 DISCUSSION

We characterized the pressure waveforms in the main and branch pulmonary arteries in individuals with and without pulmonary hypertension. Type C pressure waveforms dominated in controls and type A waveform dominated in PAH patients, while there was a mixture of types A, B and C among CTEPH patients (pre and post‐PEA). When restricted to PH patients with 0 ≤ AI ≤ 50%, we found that AI was greater and inflection time shorter in pre‐PEA CTEPH patients compared to PAH patients, consistent with previous studies (Castelain et al., 2001; Nakayama et al., 2001).

Furthermore, we addressed the accuracy of the parameters derived from PWA as a measure of arterial wave reflection and stiffness by comparing them to gold‐standard analyses that incorporate both pressure and velocity data. AI was poorly correlated to the local wave speed or WRI derived from WIA or *P*_b_/*P*_f_ derived from WSA. The inflection time did not correspond well to the time of peak BCW. In individuals with type C waveforms, the inflection point correlated more closely to the time of peak FDW than the time of the reflected wave.

### 4.1 Challenges of performing PWA in the pulmonary artery

Correct application of PWA depends on reliable identification of an inflection point in the pressure waveform, which, as we and other investigators (Castelain et al., 2001; Grignola et al., 2011) have noted, can be challenging. The normal pulmonary vasculature is presumed to be constructed in such a way that it impedes backward wave transmission and thereby minimizes the right ventricular workload (Womersley, 1958). This may explain the smooth upstrokes of the pressure waveforms with virtually non‐identifiable inflection points prior to peak systolic pressure in control subjects, consistent with the minimal BCW and *P*_b_. Furthermore, in the pulmonary circulation, there is some evidence that reflected decompression waves can be seen in healthy individuals (Dwyer et al., 2012; Hollander et al., 2001; Quail et al., 2015), which further complicates the analysis of pressure waveforms in the absence of flow velocity measurements.

We also observed that the dicrotic notch was less prominent in PH patients; it has been suggested that this may be indicative of increased vascular stiffness or right ventricular dysfunction (Kannel et al., 1981; Thyrault et al., 1998). The morphology of the pressure waveform changed from the main to branch PA in the PH patients with a more prominent systolic shoulder and greater *P*_b_ in the latter. Also, there was a better correlation between AI and wave speed and a better match between the inflection time and the timing of the peak BCW in the branch PA. Several factors could contribute to this morphological difference. Backward transmission of waves, even at a single junction, will depend on admittance matching. In the backward direction, most bifurcations are poorly matched, so retrograde transmission will be attenuated. Also, reflected waves may be out of phase, perhaps especially in CTEPH, where the lesions are commonly asymmetric; this will cause “blurring” of the reflected waves when they summate resulting in a more smooth pressure trace in the main PA.

AI > 50% was detected in several patients, e.g., in the main PA of three (27%) PAH patients. If augmentation pressure is attributed to reflected waves, an AI over 50% would be non‐physiological as it implies a reflected wave greater in magnitude than the incident wave (Baksi et al., 2016); therefore, it was assumed that the inflection points identified in these examples were implausible and they were excluded from the sub‐analyses. A late systolic inflection point (type C pressure waveform) was observed in many patients, e.g., in nearly all the controls. Type C pressure waveforms are assumed by some to be caused by late arrival of reflected waves due to longer travel distance or low wave speed (i.e., compliant arteries; O'Rourke et al., 2001). However, in systemic arteries, type C pressure waveforms can also result from failure to identify a reflection occurring in early systole that merges with the incident FCW (Hughes, Park, et al., 2013). In this case the inflection point on the pressure waveform will correspond to the second systolic peak (SBP2; Heusinkveld et al., 2019). This is consistent with our observation that *T*_i_ correlated well to the timing of the FDW in patients with type C pressure waveform, since FDW follows shortly after the second systolic peak. Type C pressure waveforms were also detected in PH patients, notably in the main PA of over 50% of the CTEPH patients. Nakayama and colleagues (Nakayama et al., 2001) observed type C pressure waveforms in almost all of the PAH patients and approximately half of the CTEPH patients and this has been interpreted as late arrival of reflected waves. However, this is inconsistent with the rapid decrease in velocity and the arrival of a substantial BCW in early systole (Figure 2a) as observed in our cohort. Furthermore, given the high wave speeds in PH patients (Ibrahim et al., 2011; Kopec et al., 2013; Su et al., 2017) and the relatively short length of the pulmonary circulation (Singhal et al., 1973), it is implausible that reflected waves will arrive late in the cardiac cycle. We believe that it is more likely that in these individuals the reflected wave is not discernible as an inflection point in the systolic upstroke; this is borne out by our observations using WIA and WSA. A similar failure to identify an early systolic inflection point has been observed in the radial artery (Kohara et al., 2005; where the measurement site is close to the reflection site) despite there being a large BCW (Zambanini et al., 2005).

### 4.2 Using PWA to assess wave reflection in PAH and CTEPH

In control subjects, wave reflection was practically negligible (WRI of 3%–6%), therefore, AI and *T*_i_ should be interpreted with caution. For this reason, subgroup analysis were performed excluding controls. Two small studies (Castelain et al., 2001; Nakayama et al., 2001) have previously reported a greater AI and shorter time to inflection point in CTEPH patients compared to PAH patients. This was interpreted as larger and earlier wave reflection and attributed to the presence of emboli in the proximal arteries in CTEPH in contrast to a more distant reflection site in PAH, which is assumed to be a distal vascular disease. Therefore, we have only included the CTEPH patients that were suitable for surgery in the current study, as they were assessed to primarily have proximal vascular lesions. Including the three inoperable patients (that either had limited radiographic signs of proximal vascular lesions or had a severe degree of peripheral vascular disease as well) in the analyses, showed the same trends, however. Among patients with 0 ≤ AI ≤ 50%, we found greater AI and shorter *T*_i_ in pre‐PEA CTEPH patients compared to PAH patients. Following PEA, AI decreased and *T*_i_ increased in the main PA. However, whether these findings imply larger and earlier wave reflection caused by proximally located thrombi and/or stiffer artery in pre‐PEA CTPEH patients is doubtful. A similar degree of wave reflection and a longer reflection time (longer Δ*T* and *T*_Pb_) were observed in pre‐PEA CTEPH patients compared to PAH patients using WIA and WSA. Also, as previously shown (Su et al., 2017), the wave speed and arterial compliance were similar in the two groups and the decrease in WRI and *P*_b_/*P*_f_ was negligibly small after PEA. Our observations therefore suggest that use of PWA to estimate the reflection magnitude and site in the pulmonary circulation may be unreliable. In general, there was a poor correlation between AI and measures of arterial stiffness and wave reflection derived from WIA and WSA, particularly in the main PA. This was the case even when analyses were restricted to PH patients with 0 ≤ AI ≤ 50%.

A poor correspondence between AI and measures of wave reflection is not surprising. In the systemic circulation, the contour of the pressure waveform and pulse pressure amplification alters with heart rate (Wilkinson et al., 2000), body height (Smulyan et al., 1998), aging (Kelly et al., 1989), arterial stiffness, geometry of the arterial system (Fok et al., 2014) and recent hemodynamic modelling studies indicate that myocardial shortening velocity and arterial stiffness are also important determinants of aortic AI (Heusinkveld et al., 2019). Our data in the pulmonary circulation are consistent with findings in the systemic circulation indicating that reliable analysis of local arterial stiffness and wave reflection requires measurement of pressure and flow (Hughes, Park, et al., 2013; Segers et al., 2007, 2017). Further studies are warranted to investigate other uses for PWA in the pulmonary circulation and whether PWA could be useful in assessing pulmonary hemodynamics in animal models of pulmonary hypertension.

## 5 LIMITATIONS

The major limitation of the present study and previous studies that employed PWA in the pulmonary artery (Castelain et al., 2001; Karamanoglu et al., 2007; Nakayama et al., 2001) is the small sample size. Therefore, the findings should be interpreted cautiously. It was not possible to investigate the influence of gender, age, and drugs through multivariable analysis and we cannot exclude the possibility that these factors could influence our observations.

Ensemble averaging waveform data was necessary to minimize noise and artefacts in the waveforms especially for velocity data collected from PH patients, where flow has been shown to be highly disturbed. Ensemble averaging pressure data might obscure inflection points; however, this approach is widely used and inspection of individual waveforms suggested this was not a problem.

## 6 CONCLUSION

Pulmonary arterial pressure waveforms differed between individuals without pulmonary vascular disease and patients with PAH and CTEPH. However, PWA is unreliable in the pulmonary circulation due to the challenges related to reliable identification of an inflection point and indices derived from PWA may not be indicative of wave reflection or arterial stiffness, particularly when type C pressure waveforms are common. Assessing wave reflection in the pulmonary artery based on PWA should therefore be discouraged.

## CONFLICT OF INTEREST

None.

## AUTHOR CONTRIBUTION

J.S., A.D.H., U.S., and C.M designed the study; J.S., L.S.H., C.M. and S.M. acquired the data; J.S. and A.D.H. analyzed the data; J.S., A.D.H., U.S. and L.S.H. interpreted the results and J.S. drafted the manuscript. All the authors have contributed to revising the manuscript critically for intellectual content and approved the final version.
